# Congenital Complete Heart Block and Post-Partum Focal Left Ventricular Dysfunction

**Published:** 2009-05-15

**Authors:** Lakshmi N Ravipati, Samir Saba

**Affiliations:** Cardiovascular Institute of the University of Pittsburgh Medical Center, Pittsburgh, Pennsylvania

**Keywords:** congenital complete heart block, post-partum left ventricular dysfunction

## Case Report

A 24 year old woman presented in her 6th month of her first pregnancy with light headedness and dizziness at rest and imbalance during walking of 2 weeks duration. Her pregnancy has been thus far uncomplicated. Prior to her pregnancy she had been in good health with no medical history or cardiac problems. She was very active, working full time, swimming, and playing softball. On her review of system, the patient reported that her heart rate had always been slow even after strenuous physical activity but denied ever seeing a cardiologist or having an electrocardiogram (ECG). Her family history was negative for any cardiac or connective tissue disorders.

Upon presentation, her ECG demonstrated second degree Mobitz type 1 atrioventricular (AV) block with intermittent complete heart block and a ventricular rate of 32 beats per minute. She underwent an echocardiogram which ruled out the presence of any structural cardiac abnormalities. Lyme titers were negative. A presumptive diagnosis of congenital high grade AV block was made.

**Figure 1:**
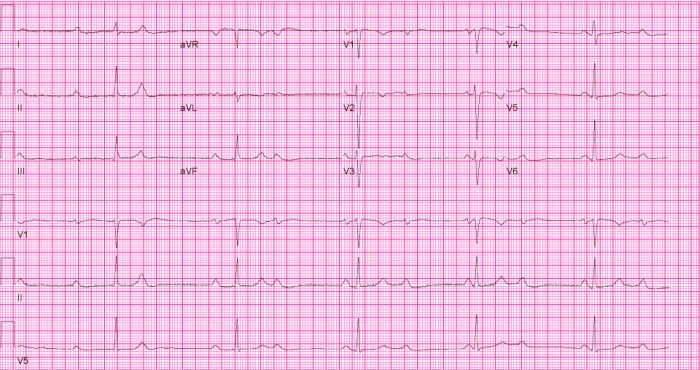
Patient's 12-lead electrocardiogram upon presentation showing complete atrioventricular block, and a junctional escape rhythm at 35 beats per minute.

Because she was minimally symptomatic, no further testing was done. She went on to deliver a health baby boy and presented again about 3 months later with chest discomfort, lightheadedness and dizziness but no syncope. Her ECG at that time showed complete heart block with narrow QRS AV nodal escape rhythm at a rate of 30 beats per minute (Figure 1). In view of her symptoms and significant bradycardia, a dual-chamber pacemaker was implanted endovascularly. The patient was discharged from the hospital feeling significantly improved.

Three days later, she presented again with pleuritic chest pain. Her pacemaker was interrogated and found to be functioning normally with no extracardiac skeletal muscle stimulation at high outputs in both the atrial and ventricular channels. In the electrophysiology laboratory, the pacing leads were inspected under fluoroscopy and were both found to be within the cardiac silhouette. An echocardiogram showed no pericardial effusion but revealed a hypokinetic left ventricular distal septum, anterior wall, and apex with an ejection fraction of 45-50 %. As the chest discomfort continued, a CT angiography of her coronary arteries was performed which showed no disease, but revealed a possible microperforation of the right ventricular lead. The patient was taken back to the electrophysiology laboratory where her right ventricular lead was repositioned in the high interventricular septum. Within 24 hours of the procedure, the patient's symptoms of pleuritic chest pain resolved. Serial echocardiograms up to 6 months after her pacemaker implantation, showed persistence of the area of septal, apical, and anterior hypokinesis with mild decrease in systolic function.

## Discussion

We present a case of a patient who presented 6 months into her first pregnancy with complete heart block presumably from congenital heart block that may have gone unnoticed for the first 24 years of her life, and who was also diagnosed with an apical cardiomyopathy 3 months after delivering her baby. This case raises a number of interesting questions. First, are the complete heart block and the cardiomyopathy two separate entities that happened to coexist in this young lady or are they related in the sense that the complete heart block could have been an early manifestation of the cardiomyopathy? Second, what is the mechanism of the cardiomyopathy in this patient? Is it a stress-related apical hypokinesis [1] related to her delivery or rather a post-partum cardiomyopathy [2] or some other form of myocarditis exacerbated by pacing manifesting mainly in the apical region of the left ventricle? Last, could the regional wall motion abnormality be secondary to the perforated right ventricular lead causing dyssynchrony in apical contraction?

Unfortunately, there is no test that can be done to ascertain the answer to these important questions. The fact that the patient had always had slow heart rates and that she was minimally symptomatic at presentation supports a diagnosis of congenital complete heart block (3, 4) particularly that the Lyme titers were negative. The level of the AV block is presumably in the AV node, as suggested by the narrow escape QRS complex, is also consistent with congenital heart block.

The etiology of the cardiomyoathy is also difficult to confirm. What is clear is that at the time of her initial presentation, 6 months into her pregnancy, the patient had a normal left ventricular function without any regional wall motion abnormalities. Three months after delivering, she had evidence of an apical hyopkinesis which persisted for more than 6 months after delivering the baby and for about 3 months after it was originally detected. This time course is inconsistent with the diagnosis of Takotsubo in which all regional wall motion abnormalities resolve usually within 2 weeks of the stressful event. The fact that the cardiomyopathy was not present 6 months into the pregnancy and was diagnosed after delivery in the context of looking for any evidence of pericardial effusion is suggestive of an unusual form of focal myocarditis or postpartum cardiomyopathy. If this, in fact, is the case, then this finding suggests that possibly many cases of mild post-partum cardiomyopathy or myocarditis are missed after otherwise uneventful pregnancies and deliveries.

Finally, although pacing of the heart alters the electrical and therefore the mechanical activation of various parts of the ventricles, it almost never causes a regional wall motion abnormality that persists for months after the lead position is changed.

In summary, we present a case of complete heart block of probable congenital etiology in a pregnant 24 year old patient who developed a new apical left ventricular dysfunction after delivering her baby. Although cases of congenital heart block and of left ventricular dysfunction developing in the post-partum period have been reported in the literature, to our knowledge, this is the first case of both these two entities coexisting in the same patient. Common clinical or genetic predispositions to these two findings may exist but cannot be ascertained at the present time.
